# Competition between Sliding and Peeling of Graphene Nanoribbons under Horizontal Drag

**DOI:** 10.3390/ma15093284

**Published:** 2022-05-04

**Authors:** Ruiyang Li, Fan Xu

**Affiliations:** 1 Institute of Mechanics and Computational Engineering, Department of Aeronautics and Astronautics, Fudan University, 220 Handan Road, Shanghai 200433, China; 20110290002@fudan.edu.cn; 2 Zhangjiang Fudan International Innovation Center, Fudan University, Shanghai 200433, China

**Keywords:** graphene nanoribbon, nanocontact, peeling, sliding

## Abstract

In the process of graphene nanoribbons’ (GNRs) preparation and measurement, mechanical methods such as lifting and dragging are inevitably used to move GNRs, and manipulation of GNRs using these approaches results in intriguing responses such as peeling and sliding. Understanding the mechanical behaviors of GNRs is crucial for the effective use of mechanical deformation as a tool for the measurement and characteristics of low-dimensional material properties. Here, we explore intricate coupling behaviors of peeling and sliding of GNRs under horizontal drag. Using molecular dynamics simulation, we explore effects of lifting height, dragging velocity, length, and orientation of GNRs on mechanical behaviors. We reveal a competition between sliding and peeling of GNRs under horizontal drag and provide a phase diagram. The peeling behavior is found to be originated from the decrease of sliding velocity caused by the sinking of tail atoms. The results not only advance our insightful understanding of the underlying mechanism of different mechanical responses of GNRs but may also guide the precise manipulations of nano surfaces and interfaces.

## 1. Introduction

As a kind of carbon nanomaterial, graphene nanoribbons (GNRs) have attracted considerable attention in recent years. In addition to excellent physical and mechanical properties of conventional graphene [1,2,3,4,5], GNRs have even more unique properties due to its huge aspect ratio [6]. Therefore, GNRs have been widely used in nanoelectronics [7,8], biosensing [9], electrode materials [10], and other aspects. Due to their two-dimensional characteristics, many important properties are related to the surface and interface. Kawai et al. [11] investigated the sliding behavior of GNRs on a gold substrate and observed superlubrication experimentally. The origin of superlubrication is the van der Waals interaction at the GNRs/substrate interface. Later, Gigli et al. [12] studied the peeling (vertically lifting) behavior of GNRs on a gold substrate, and revealed that the intricate peeling behavior is mainly caused by the mixed-mode of friction and adhesion at the interface. Chang et al. [13] proposed a way to induce graphene self-folding using atomic force microscope (AFM) for surface morphology control of GNRs. Ouyang et al. [14] numerically predicted serpentine movement of narrow GNRs on hexagonal boron nitride (hBN) substrates and explored the effect of nanoserpent morphology on friction. Savin et al. [15] studied the twistons structure of GNRs and showed that the contact between GNR and substrate would alter their physical and mechanical properties. Recently, Xue et al. [16] further explored rich dynamics in peeling and sliding of GNRs based on the continuum model and finite element method. While complex mechanical responses may occur during GNR manipulation, intriguing interactions between different mechanical responses remain elusive and have not been fully understood.

Here, we explore the coupling behavior of peeling and sliding of GNRs on a gold substrate under horizontal dragging, based on molecular dynamics with a variety of initial conditions. We reveal that, when the lifting height of GNRs remains relatively small, horizontal dragging of the lifting end of GNRs keeps the geometric configuration unchanged, and makes GNRs purely slide on the substrate, while, when the lifting height becomes larger, the motion of GNRs turns out to be peeling. Such sliding/peeling transition implies an intricate competition in between. We therefore examine the lifting height, dragging velocity, and length of GNRs on the sliding/peeling interplay and provide a phase diagram. We find that the sinking of tail atoms is a stable geometric configuration, leading to the decrease of sliding velocity of GNRs, and thus yields the peeling response. Understanding the sliding and peeling behaviors of GNRs may help the effective manipulation of mechanical motion as a tool for measurements and characteristics of low-dimensional material properties and could guide the assembly of nanostructures such as GNRs arrays used in optics [17,18,19].

## 2. Model and Method

**Figure 1:**
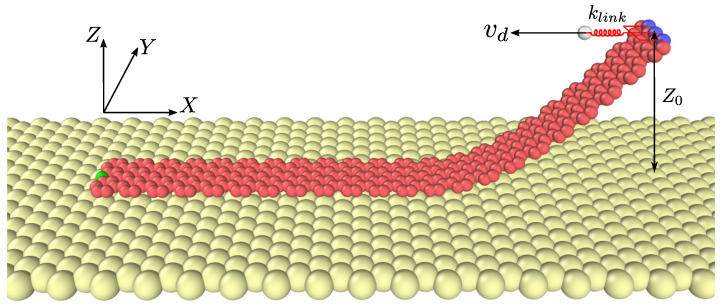
Schematic of the computational model. GNR is horizontally dragged by imposing a speed $v_{d}$ to the virtual atom connecting to the atoms (in blue color) at the lifted side, using a linear spring of stiffness $k_{link}=1.5$ N/m. The lifting height $Z_{0}$ remains unchanged during the dragging process, and we record the motion of the green atom.

We explore sliding and peeling of GNRs by dragging lifted side with a constant speed $v_{d}$ on a gold substrate (see Figure 1). In molecular dynamics simulations, we set the width of GNRs ∼0.7 nm and the length $l_{x}$ varying from 9.2 nm to 20 nm, which are chosen within the scope of experiments [11]. For the gold substrate, we choose the Au (111) surface with the reconstruction that has been observed in vacuum [20], and all Au atoms are fixed. For the initial position of GNR relative to the substrate lattice structure, the GNR long axis lies parallel to the Au [1, −2, 1] crystallographic direction. To examine the effect of lifting height on mechanical behavior, we vary the height of lifted side $Z_{0}=1∼3$ nm.

The simulation is divided into two steps, i.e., lifting process and dragging one. We first lift the right side of a relaxed GNR by applying speed in the *Z* direction (atoms highlighted in blue color). When the targeted height $Z_{0}$ is achieved, the GNR is relaxed again in the lifted configuration. During the dragging process, we connect the atoms (blue ones) in the lifted side to a virtual atom using a linear spring of stiffness $k_{link}$. The virtual atom is given a range of constant velocity from $v_{d}=0.1$ to 0.5 m/s in the negative *x* direction. While dragging, we record the motion $U_{x}$ and $U_{z}$ of the atom (in green color) at the tail of GNR.

We use the reactive empirical bond order (REBO) force field [21] to model the intralayer C–C interactions. Non-bonded interlayer interactions between C and Au atoms are modeled by standard Lennard–Jones (LJ) potential [22]: $V_{LJ}^{12-6}\left(r\right)=4\varepsilon \left[{\left(\sigma /r\right)}^{12}-{\left(\sigma /r\right)}^{6}\right]$, where the parameters ε=2.5 meV, σ=2.74 Å, and cutoff length $L_{cutoff}=10$ Å [11], so as to limit LJ atom pair calculations to short range interactions. The interaction of hydrogenated edges of GNRs with Au substrate is not explicitly taken into account, i.e., the model does not involve hydrogen atoms in Figure 1. However, the empirical LJ parameters based on fitting to the experimental data of Kawai et al. [11] imply accounting of this interaction. We apply LAMMPS packages [23] for simulations with an initial temperature T=4.8 K used in experiments [11]. We use a Nose–Hoover thermostat in the relaxing process, and obey Langevin dynamics in the lifting and dragging process with damping parameter $\gamma_{L}=0.01$ ps.

## 3. Results and Discussion

### 3.1. Competition between Peeling and Sliding Behaviors under Horizontal Drag

**Figure 2:**
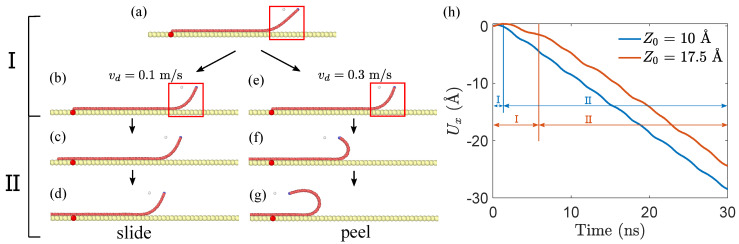
Schematic of sliding and peeling of GNRs. (**a**) the initial geometry of GNR with $l_{x}∼$ 9.2 nm and lifting height $Z_{0}=15$ Å before dragging. The red atom in the substrate refers to a reference point. (**b**–**d**) show the sliding-dominant process when $v_{d}=0.1$ m/s, while (**e**–**g**) demonstrate the peeling-prevailing progress when $v_{d}=0.3$ m/s. The motion of GNRs can be distinguished into two stages: in stage I, the main movement is constrained to the lifted part (within red box), while, in stage II, the motion involves both sliding and peeling. (**h**) shows the displacement–time diagram of GNRs with $l_{x}∼$ 11.3 nm, $v_{d}=0.1$ m/s and $Z_{0}$ = 10, 17.5 Å.

We investigate the peeling and sliding responses of GNRs under horizontal drag. As illustrated in Figure 2, different initial conditions may lead to different behaviors, i.e., sliding or peeling. The GNR configuration remains almost unchanged in the sliding response, while the shape dramatically alters in the peeling process. For both sliding and peeling, one can distinguish the motion into two stages, i.e., I and II, according to time sequence (see Figure 2). In stage I, the main motion of GNR is limited to the shape change of the lifted part (within red box), while, in stage II, the GNR exhibits global deformation and motion. More precisely, for the sliding-dominant process (see Figure 2d) in stage II, the displacements along the *x* direction of all GNR atoms equals that of the virtual atom, while, for the peeling-dominant process (see Figure 2g) in stage II, the displacement is smaller than that of the virtual atom. This distinction allows us to quantify the difference between sliding and peeling behaviors. We take the tail atom (in green color) shown in Figure 1 as the representative one, and distinguish between sliding and peeling responses by comparing the displacements of tail and virtual atoms. In a displacement–time diagram (see Figure 2h), stage I can be identified by the slope of the curve that varies from positive to negative.

**Figure 3:**
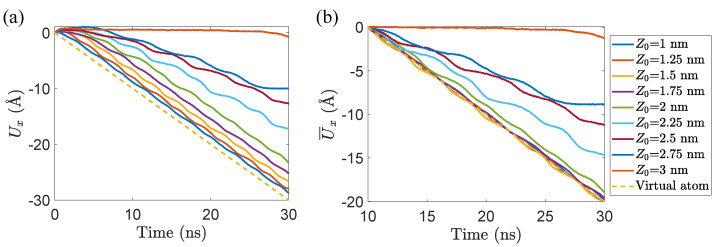
Displacement–time diagram of tail and virtual atoms of GNR with $l_{x}∼$ 9.2 nm, $v_{d}=0.1$ m/s and different lifting heights $Z_{0}$. (**a**) considers the whole simulation time, including both stages I and II; (**b**) plots ${\bar{U}}_{x}$ by choosing 10 ns as the zero displacement reference point, only involving stage II.

Figure 3 plots displacement–time diagrams of the tail atom and the virtual one. It can be seen that the absolute values of displacement of the tail atom decrease with the rise of lifting height $Z_{0}$. The displacement–time curves of tail atom in five cases whose $Z_{0}$ ranges from 1 to 2 nm and of the virtual atom are overlapped in Figure 3b, which indicates that the movement of GNR in these cases is sliding rather than peeling. However, in Figure 3a, the displacement curves of these five cases do not coincide, which is caused by the motion in stage I. With the rise of lifting height, the proportion of the stage I increases (see Figure 2h and Figure 3a). As mentioned before, in stage I, the main motion is constrained to the lifted side so that the tail atom is basically stationary in stage I. The more time the stage I takes up, the smaller the absolute values of the displacement $U_{x}$ are. This is why the absolute values of $U_{x}$ decrease with the rise of lifting height $Z_{0}$ even in the cases when GNR is sliding. Therefore, to clearly distinguish sliding from peeling, comparison of the displacement of tail and virtual atoms should begin after GNR is dragged for a period of time, so as to avoid the influence of stage I. In our simulations, 10 ns is sufficient to cover stage I and thus we choose this point as the zero displacement reference and record a relative displacement ${\bar{U}}_{x}$.

**Figure 4:**
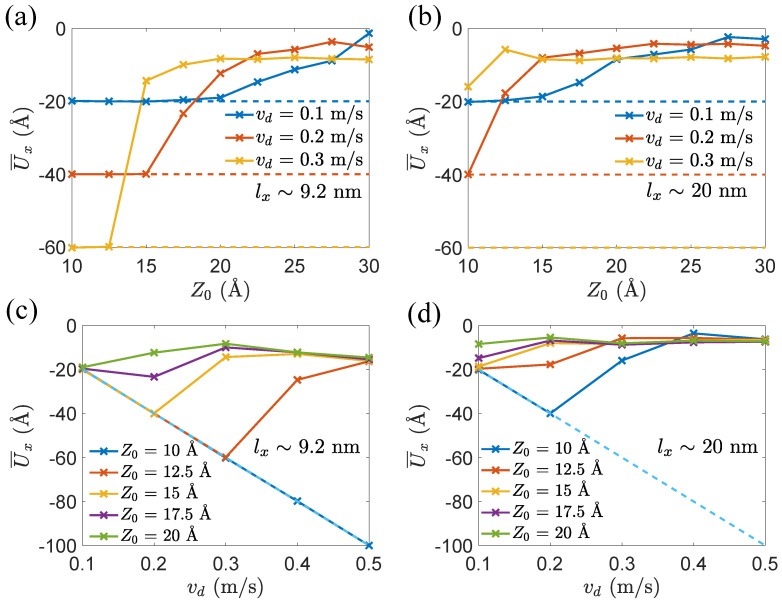
(**a**,**b**) Variations of displacement ${\bar{U}}_{x}$ of tail atom with respect to lifting height $Z_{0}$ for different dragging velocities $v_{d}$ at time of 30 ns. Dotted lines represent the displacements of virtual atom; (**c**,**d**) variation of displacement ${\bar{U}}_{x}$ of tail atom with respect to the dragging velocity $v_{d}$ for different lifting heights $Z_{0}$ at time of 30 ns. Dotted lines represent the displacements of virtual atom.

With such a way to distinguish between sliding and peeling responses, we explore the effects of lifting height $Z_{0}$, dragging velocity $v_{d}$, and length $l_{x}$ on the movement and deformation of GNR being lifted and dragged. We examine two GNRs with $l_{x}∼$9.2 nm and $l_{x}∼$20 nm, respectively, selected as the representatives of short and long GNRs. In Figure 4, solid curves show how ${\bar{U}}_{x}$ varies with respect to $Z_{0}$ at different dragging velocities $v_{d}$, while dotted lines indicate the displacements of the virtual atom. When both curves become overlapped, GNR is sliding; otherwise, GNR is peeling. The more space between both curves, the greater the degree of peeling. In addition, one can see that, with the rise of $Z_{0}$, the behavior of GNR changes from sliding to peeling, as also revealed in Figure 3. Moreover, when the dragging velocity $v_{d}$ increases, the translation from sliding to peeling occurs earlier. Comparing Figure 4b with Figure 4a, one can see that, when the GNR becomes longer, peeling behavior prevails, which suggests that it is easier to peel a long GNR under horizontal drag. Note that, when the lifting height $Z_{0}$ reaches a certain value (e.g., $Z_{0}=20$ Å in Figure 4b, continuing to increase $Z_{0}$ does not influence the degree of peeling response.

We next focus on the effect of dragging speed $v_{d}$. According to Figure 4c, when the lifting height $Z_{0}>10$ Å, with the increase of dragging velocity $v_{d}$, the behavior of GNR changes from sliding to peeling. While the motion is peeling, increasing $v_{d}$ has a limited effect on ${\bar{U}}_{x}$. A similar mechanism is observed in a long GNR with $l_{x}∼$ 20 nm (see Figure 4d). After the behavior of GNR alters from sliding to peeling, continuously increasing $v_{d}$ and $Z_{0}$ will not significantly affect ${\bar{U}}_{x}$.

**Figure 5:**
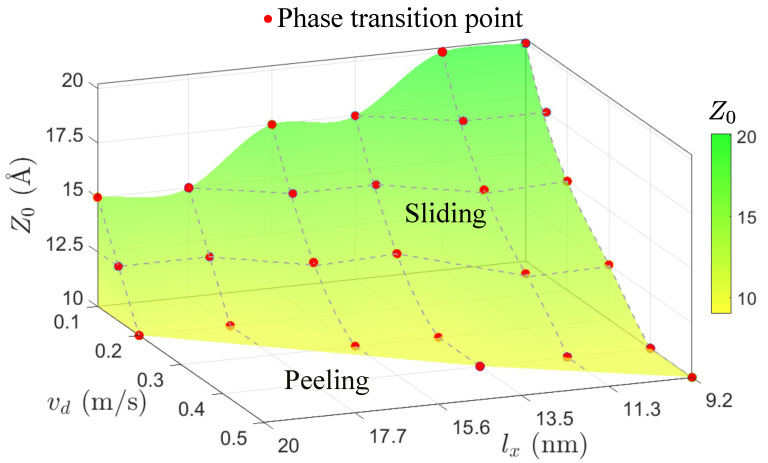
Phase diagram of sliding and peeling responses. With the rise of $l_{x}$ and $v_{d}$, peeling behavior becomes energetically favorable.

To provide an overall view of sliding and peeling responses of GNRs under horizontal drag, we construct a phase diagram in Figure 5 (also see Supplementary Video S1). With the increase of $l_{x}$ and $v_{d}$, peeling behavior becomes energetically favorable, which is mainly attributed to the van der Waals adsorption at the GNRs/substrate interface. According to Kawai et al. [11], GNR shows a superlubricity characteristic on the gold substrate, which implies that the friction does not significantly increase with the rise of $l_{x}$. Therefore, the friction may not be the reason for the fact that the longer GNRs are easier to be peeled. We suspect that, when GNR gets longer, the tail becomes further away from the lifting part and thus is less affected by the drag, so it is more difficult to move.

### 3.2. The Origination of Peeling Response

**Figure 6:**
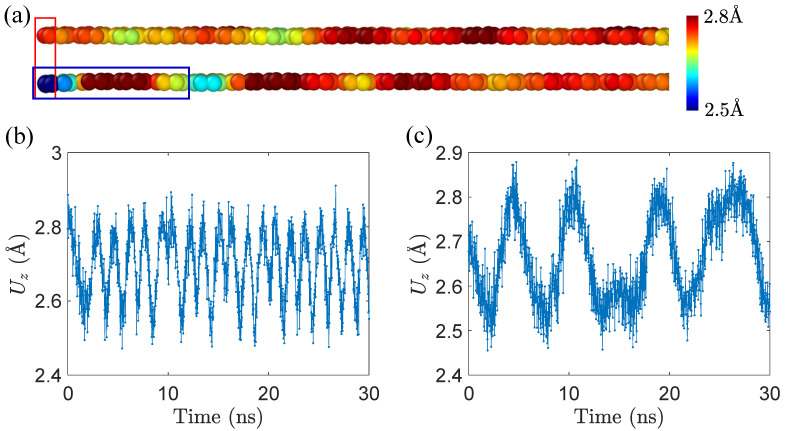
The sinking of tail atoms. (**a**) two morphologies of tail atoms in GNRs under horizontal drag. The upper one is in the sliding process, while the lower one is in the peeling process. The color bar shows the distance from the substrate. We compute the LJ potential energy of atoms in the blue box that forms a moiré pattern. (**b**,**c**) variations of $U_{z}$ of tail atom in a GNR with $l_{x}∼$ 20 nm. (**b**) $v_{d}$ = 0.2 m/s and $Z_{0}$ = 10 Å, sliding behavior; (**c**) $v_{d}$ = 0.4 m/s and $Z_{0}$ = 17.5 Å, peeling response.

Mechanical responses of GNRs are closely related to their morphologies. Figure 6 demonstrates two morphologies of GNR tail in the *z* direction. The upper one in Figure 6a is in the sliding process, while the lower one shows the morphology when the tail sinks in the peeling stage. We plot $U_{z}$-time diagrams in Figure 6b,c under sliding and peeling responses, respectively. One can see that, for GNR in the sliding state, the $U_{z}$-time curve shows periodic short-wavelength undulation, while, in the peeling state, the curve illustrates irregular long waves, and stays in the valley longer than the peak as a result of the sink of tail atoms.

**Figure 7:**
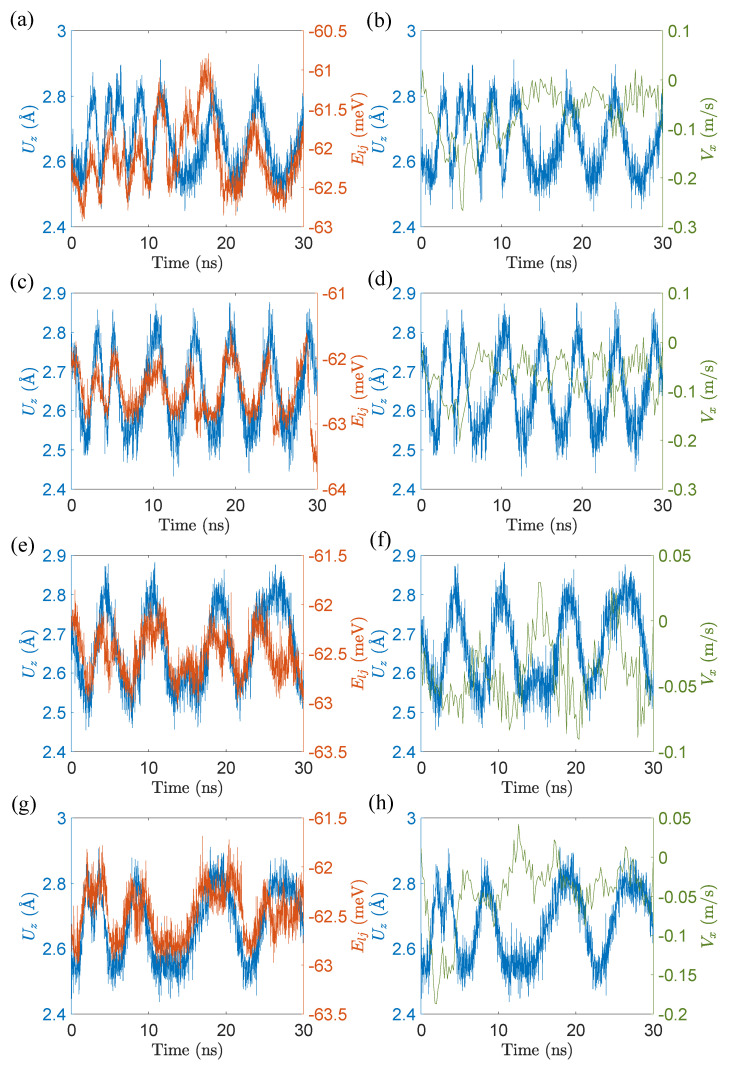
Variations of displacement $U_{z}$, velocity $V_{x}$ of tail atom, and LJ potential energy $E_{lj}$ of the moiré pattern in GNRs under horizontal drag. (**a**,**b**) $l_{x}∼$ 9.2 nm, $v_{d}$ = 0.3 m/s and $Z_{0}$ = 17.5 Å; (**c**,**d**) $l_{x}∼$ 11.3 nm, $v_{d}$ = 0.4 m/s and $Z_{0}$ = 17.5 Å; (**e**,**f**) $l_{x}∼$ 20 nm, $v_{d}$ = 0.3 m/s and $Z_{0}$ = 12.5 Å; (**g**,**h**) $l_{x}∼$ 9.2 nm, $v_{d}$ = 0.4 m/s and $Z_{0}$ = 17.5 Å. All the GNRs exhibit peeling behavior.

To further explore the effect of this phenomenon on the peeling behavior, we calculate the time variation of LJ potential energy $E_{lj}$ of the moiré pattern and the average velocity (every 0.2 ns) $V_{x}$ of the tail atom in the *x* direction. Figure 7 illustrates that the variation of $E_{lj}$ over time is nearly consistent with the undulation of $U_{z}$, which suggests that the geometry of tail atoms in the sinking state holds a lower energy state and is thus more stable. In addition, we reveal that the absolute value of $V_{x}$ declines when $U_{z}$ decreases. Combining the two points above leads to the following statement—it is the long time sinking of the tail atoms that causes the peeling behavior. During the peeling process, the tail swings up and down irregularly and sinks for a while. This is because the sinking geometry is energetically more stable, leading to the slow down of $V_{x}$, which then results in a velocity difference between the lifting part and the tail that eventually triggers the peeling behavior.

### 3.3. The Influence of Orientation

**Figure 8:**
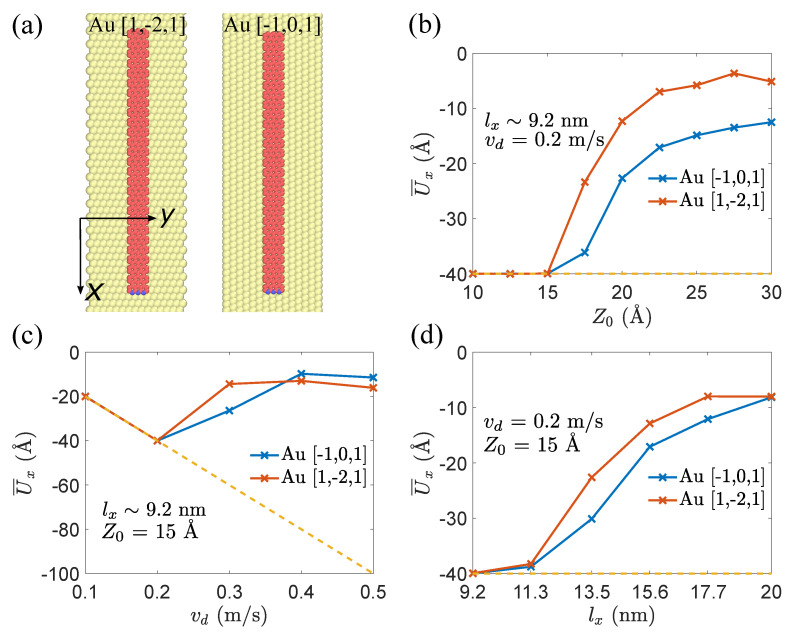
(**a**) Schematic of two orientations, GNRs on Au [1, −2, 1] and Au [−1, 0, 1] crystallographic directions; (**b**–**d**) variation of displacement ${\bar{U}}_{x}$ of tail atom for different orientations with respect to dragging velocity $v_{d}$, lifting height $Z_{0}$, and length $l_{x}$ at time of 30 ns. Dotted lines represent the displacements of virtual atom.

For the GNR-Au heterojunction system, the orientation of GNR on the Au substrate affects the adsorption energy between GNR and Au substrate [24], which may lead to changes in the mechanical responses of GNR. To explore the effect of orientation of GNR, we choose a typical orientation, i.e., GNR is parallel to the Au [−1, 0, 1] crystallographic direction. This orientation rotates the previous Au substrate by $30^{\circ }$(or equivalently $90^{\circ }$), as shown in Figure 8a. We examine the effect of lifting height $Z_{0}$, dragging velocity $v_{d}$, and length $l_{x}$ on the sliding and peeling responses of GNRs. As shown in Figure 8b–d, orientation effect is mainly manifested under peeling behaviors. With the same initial conditions, different orientations lead to different displacements ${\bar{U}}_{x}$ in the peeling response. In the considered cases, the displacement ${\bar{U}}_{x}$ of Au [−1, 0, 1] is less than that of Au [1, −2, 1] crystallographic direction, while the orientation does not change the phase transition points.

## 4. Conclusions

We have investigated the sliding and peeling behaviors of GNRs under horizontal drag. By comparing the displacement of the virtual atom with that of the tail atom, one can quantify the distinction between sliding and peeling behaviors. In addition, we have explored the effects of initial conditions on mechanical responses, and revealed that the motion of GNRs can change from sliding to peeling with the increase of lifting height $Z_{0}$, dragging velocity $v_{d}$, and length $l_{x}$. We have shown that the orientation of GNRs on the Au substrate does not change phase transition points between sliding and peeling behaviors. Moreover, we have provided a phase diagram of sliding and peeling behaviors, and revealed that the origination of the peeling response comes from the long-time sinking of the tail atoms. Our results not only contribute to the insightful understanding of origin and mechanism of different mechanical responses of GNRs but may also guide the precise nanomanipulations of low-dimensional interfaces.

## Data Availability

Not applicable.

## Supplementary Materials

The following supporting information can be downloaded at: https://www.mdpi.com/article/10.3390/ma15093284/s1, Video S1: Competition between sliding and peeling of graphene nanoribbons under horizontal drag.

## Abbreviations

The following abbreviations are used in this manuscript:

GNR	Graphene nanoribbon
AFM	Atomic force microscope
hBN	Hexagonal boron nitride
REBO	Reactive empirical bond order

## References

[1] Zhao H., Min K., Aluru N.R. (2009). Size and chirality dependent elastic properties of graphene nanoribbons under uniaxial tension. Nano Lett..

[2] Apell S.P., Hanson G., Hägglund C. (2012). High optical absorption in graphene. arXiv.

[3] Novoselov K.S., Jiang Z., Zhang Y., Morozov S., Stormer H.L., Zeitler U., Maan J., Boebinger G., Kim P., Geim A.K. (2007). Room-temperature quantum hall effect in graphene. Science.

[4] Geim A.K., Novoselov K.S. (2007). The rise of graphene. Nat. Mater..

[5] Lee C., Wei X., Kysar J.W., Hone J. (2008). Measurement of the elastic properties and intrinsic strength of monolayer graphene. Science.

[6] Celis A., Nair M.N., Taleb-Ibrahimi A., Conrad E., Berger C., Heer W.D., Tejeda A. (2016). Graphene nanoribbons: Fabrication, properties and devices. J. Phys. D Appl. Phys..

[7] Wei D., Liu Y., Zhang H., Huang L., Wu B., Chen J., Yu G. (2009). Scalable synthesis of few-layer graphene ribbons with controlled morphologies by a template method and their applications in nanoelectromechanical switches. J. Am. Chem. Soc..

[8] Sazonova V., Yaish Y., Üstünel H., Roundy D., Arias T.A., McEuen P.L. (2004). A tunable carbon nanotube electromechanical oscillator. Nature.

[9] Min S.K., Kim W.Y., Cho Y., Kim K.S. (2011). Fast DNA sequencing with a graphene-based nanochannel device. Nat. Nanotechnol..

[10] Lin J., Peng Z., Xiang C., Ruan G., Yan Z., Natelson D., Tour J.M. (2013). Graphene nanoribbon and nanostructured SnO2 composite anodes for lithium ion batteries. ACS Nano.

[11] Kawai S., Benassi A., Gnecco E., Söde H., Pawlak R., Feng X., Müllen K., Passerone D., Pignedoli C.A., Ruffieux P. (2016). Superlubricity of graphene nanoribbons on gold surfaces. Science.

[12] Gigli L., Kawai S., Guerra R., Manini N., Pawlak R., Feng X., Müllen K., Ruffieux P., Fasel R., Tosatti E. (2018). Detachment dynamics of graphene nanoribbons on gold. ACS Nano.

[13] Chang J.S., Kim S., Sung H.-J., Yeon J., Chang K.J., Li X., Kim S. (2018). Graphene nanoribbons with atomically sharp edges produced by AFM induced self-folding. Small.

[14] Ouyang W., Mandelli D., Urbakh M., Hod O. (2018). Nanoserpents: Graphene nanoribbon motion on two-dimensional hexagonal materials. Nano Lett..

[15] Savin A., Korznikova E., Dmitriev S. (2020). Twistons in graphene nanoribbons on a substrate. Phys. Rev. B.

[16] Xue Z., Chen G., Wang C., Huang R. (2022). Peeling and sliding of graphene nanoribbons with periodic van der Waals interactions. J. Mech. Phys. Solids.

[17] Hu G., Zheng C., Ni J., Qiu C., Alù A. (2021). Enhanced light-matter interactions at photonic magic-angle topological transitions. Appl. Phys. Lett..

[18] Sarsen A., Valagiannopoulos C. (2019). Robust polarization twist by pairs of multilayers with tilted optical axes. Phys. Rev. B.

[19] Zhumabek T., Valagiannopoulos C. (2020). Light trapping by arbitrarily thin cavities. Phys. Rev. Res..

[20] Harten U., Lahee A., Toennies J.P., Wöll C. (1985). Observation of a soliton reconstruction of Au (111) by high-resolution helium-atom diffraction. Phys. Rev. Lett..

[21] Brenner D.W., Shenderova O.A., Harrison J.A., Stuart S.J., Ni B., Sinnott S.B. (2002). A second-generation reactive empirical bond order (REBO) potential energy expression for hydrocarbons. J. Phys. Condens. Matter.

[22] Jorgensen W.L., Chandrasekhar J., Madura J.D., Impey R.W., Klein M.L. (1983). Comparison of simple potential functions for simulating liquid water. J. Chem. Phys..

[23] Plimpton S. (1995). Fast parallel algorithms for short-range molecular dynamics. J. Comput. Phys..

[24] Yortanlı M., Mete E. (2019). Common surface structures of graphene and Au (111): The effect of rotational angle on adsorption and electronic properties. J. Chem. Phys..

